# Photoperiod-Induced Increases in Bone Mineral Apposition Rate in Siberian Hamsters and the Involvement of Seasonal Leptin Changes

**DOI:** 10.3389/fendo.2017.00357

**Published:** 2017-12-19

**Authors:** Marie Kokolski, Francis J. Ebling, James R. Henstock, Susan I. Anderson

**Affiliations:** ^1^Division of Medical Sciences and Graduate Entry Medicine, School of Medicine, University of Nottingham, Royal Derby Hospital, Derby, United Kingdom; ^2^School of Life Sciences, University of Nottingham, Nottingham, United Kingdom; ^3^Institute of Ageing and Chronic Disease, University of Liverpool, Liverpool, United Kingdom

**Keywords:** leptin, Siberian hamster, mineral apposition rate, growth plate, microCT

## Abstract

The adipokine leptin regulates energy balance, appetite, and reproductive maturation. Leptin also acts on bone growth and remodeling, but both osteogenic and anti-osteogenic effects have been reported depending on experimental conditions. Siberian hamsters (*Phodopus sungorus*) have natural variation in circulating leptin concentrations, where serum leptin is significantly decreased during the short day (SD)-induced winter state. In summer long day (LD) photoperiods, appetite and body adiposity increase with associated central leptin insensitivity. This natural change in leptin secretion was exploited to investigate leptin’s effect on bone growth. Hamsters were injected with calcium-chelating fluorescent dyes to measure bone mineral apposition rate (MAR). Measurements were initially obtained from 5-week and 6-month-old animals maintained in low leptin (SD) or high leptin (LD) states. A further study investigated effects of chronic administration of recombinant mouse leptin to hamsters housed in SD and LD conditions; growth plate thickness and bone density were also assessed. As expected, a reduction in body mass was seen in hamsters exposed to SD, confirming the phenotype change in all studies. Serum leptin concentrations were significantly reduced in SD animals in all studies. MAR was reproducibly and significantly increased in the femurs of SD animals in all studies. Vitamin D and growth plate thickness were significantly increased in SD animals at 6 months. No effect on bone density was observed in any study. Taken together these data suggest that bone growth is associated with the low leptin, winter, lean state. In leptin-treated animals, there was a significant interaction effect of leptin and photoperiod. In comparison to their vehicle counterparts, SD animals had decreased and LD animals had increased MAR, which was not apparent prior to leptin administration. In conclusion, increased MAR was associated with low serum leptin levels in early life and sustained over 6 months, implying that leptin has a negative effect on bone growth in this model. The unexpected finding that MAR increased after peripheral leptin administration in LD suggests that leptin exerts different effects on bone growth dependent on initial leptin status. This adds further weight to the hypothesis that leptin-treated LD animals display central leptin resistance.

## Introduction

Leptin is a 16-kDa peptide hormone which is synthesized and released primarily by adipose tissue (1). Circulating concentrations are positively correlated with increases in peripheral body fat (2). Leptin was first investigated for its role in appetite regulation and control of energy expenditure (3), but since then it has been discovered that leptin and its receptor are involved in regulation of reproduction, immune function, serum glucose levels, angiogenesis (4), and bone homeostasis (5).

A number of animal models have been used to understand the role of leptin in bone mass accrual, including the *ob/ob* mouse which cannot produce leptin and the *db/db* mouse which has dysfunctional leptin receptors. Initial studies by Ducy et al. (5) found *ob/ob* mice to have a high bone mass phenotype in their vertebrae and femurs and an increased bone formation rate compared to wild-type animals. In contrast, Steppan et al. (6) found that a high bone mass in the long bones of *ob/ob* mice was achieved only after administering peripheral leptin. Hamrick et al. (7) showed that contrasting bone phenotypes could also appear within the same *ob/ob* model, with lumbar vertebrae being longer and having an improved bone architecture, whereas femurs were shorter with poor bone architecture.

In the current literature, four mutations in the gene coding for leptin have been reported which lead to leptin deficiency and result in severe obesity (8–12). Leptin replacement has been investigated as a potential treatment to be used in cases of leptin deficiency (13), and so it is important to understand the effects of leptin on bone. However, leptin deficiency is rare, and it is more common to find extremely low or high levels in humans rather than a complete lack. Lipodystrophy, for example, is characterized by the loss of subcutaneous adipose tissue (14), which results in low circulating leptin levels and has been associated with human immunodeficiency virus antiviral treatments (15). Low leptin levels are also found in those suffering from anorexia nervosa and hypothalamic amenorrhea, and conversely, high circulating leptin levels are found in obesity.

The Siberian hamster, *Phodopus sungorus*, has been used extensively as a robust model of seasonal change which has been used to investigate energy expenditure, reproductive changes, and adiposity (16, 17). The Siberian hamster seasonal change is stimulated by changes in the production of melatonin, which increases during the short day (SD; winter) photoperiod and prompts metabolic and physical changes (18). Upon exposure to SD photoperiods, they reduce their food intake, even if given food *ad libitum* (19), which reduces their body weight, abdominal fat reserves, and serum leptin levels. In addition, reproductive system regression occurs in both sexes; the testes and epididymal fat pad weights of males and uterine weight of females are markedly reduced in SD-exposed animals (20), resulting in decreased levels of circulating sex hormones (21, 22). Conversely, exposure to long day (LD; summer) photoperiods increases appetite and results in lipogenesis, increased body mass, larger reproductive organs, and elevated leptin production. This comprehensive response to photoperiod provides a model to investigate bone growth and remodeling in animals with naturally high and low serum leptin levels.

Only one previous study has examined bone parameters in the Siberian hamster model and found no evidence for effects of recombinant leptin on trabecular bone mass or mechanical properties of bone; however, this study did not measure bone growth (23). The aim of this study was to use the naturally high and low leptin levels, induced by photoperiod in the Siberian hamster, as a model system to investigate the relationship between circulating leptin concentrations and bone mineral apposition rate (MAR) and bone density. Following from this, the mechanistic effect of leptin on any observed changes was investigated by supplementing animals in both photoperiodic states with peripheral leptin and measuring MAR longitudinally before and after leptin supplementation.

## Materials and Methods

### Animals

All studies were carried out in accordance with the UK Animals (Scientific Procedures) Act of 1986 (project licenses: PPL 40/3065 and PPL 40/3604) and approved by the University of Nottingham Animal Welfare and Ethical Review Board. Animals were from a colony of Siberian hamsters (*P. sungorus*) maintained at the University of Nottingham Biomedical Services Unit (20). Animals were group housed in 21°C and 40% humidity conditions and allowed *ad libitum* access to water and food (Teklad 2019 global 19% protein extruded rodent diet, Harlan, UK). Hamsters were housed in either LD conditions comprising 16 h light:8 h dark or SD conditions of 8 h light:16 h dark.

### Experimental Procedure—Study 1

**Figure 1:**
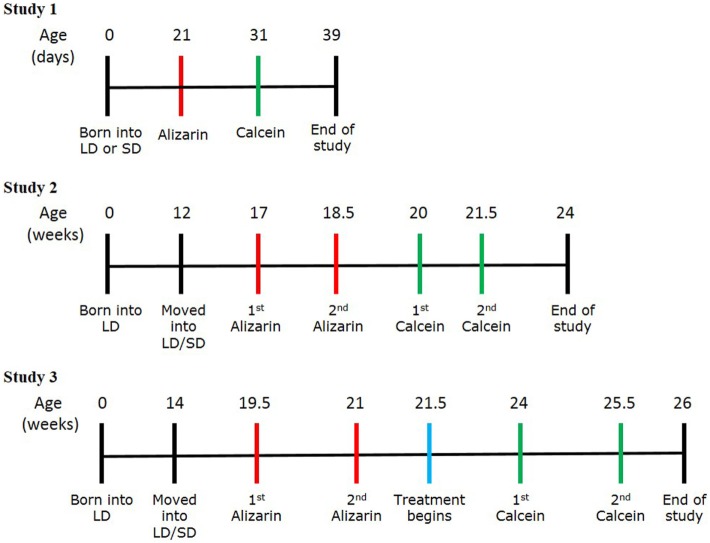
Experimental protocol schemes for studies 1, 2, and 3. Timelines showing the stages of each study, including birth, transfer into the photoperiod environments, the timings of calcein and alizarin injections, and the end of study.

Siberian hamsters (three females and one male) from mothers that were either housed in LD throughout or from mothers that had been exposed to SD as soon as pregnancy was detected by palpation, generally about 7 days before parturition (four females and two males) were studied (Figure 1). At 3 weeks, the animals were injected with 150 mg/kg of alizarin (Sigma, UK), and 10 days later they were injected with 100 mg/kg of calcein (Sigma, UK). Both dyes were diluted in 1.4% sodium bicarbonate and injected into the intraperitoneal cavity. A week later, the animals were euthanized with sodium pentobarbital (Euthatal, Merial, UK). Fore and hind limb long bones and lumbar vertebrae were removed for cortical appositional growth analysis, and serum was obtained for analysis of vitamin D and leptin levels.

### Experimental Procedure—Study 2

Male Siberian hamsters were randomly housed in LD (*n* = 6) or SD (*n* = 7) conditions from the age of 12 weeks and left to acclimatize for 5 weeks. At 17 weeks of age, all animals were injected with 75 mg/kg of alizarin, and this was repeated at 18.5 weeks (Figure 1). At 20 weeks, the animals were injected with 40 mg/kg of calcein, which was repeated at 21.5 weeks. Both dyes were diluted in 1.4% sodium bicarbonate and injected into the intraperitoneal cavity. Both dyes were administered at lower concentrations than study 1 to prevent potential distress to the animal by administering the larger volumes needed for the larger animal size. The animals were euthanized at 24 weeks using sodium pentobarbital (Euthatal, Merial, UK). The testes and testicular fat pads were removed from each animal and weighed. Lumbar vertebrae and femurs were removed for cortical appositional growth analysis, and serum was obtained for analysis of vitamin D and leptin levels.

### Experimental Procedure—Study 3

Female Siberian hamsters were randomly housed in LD or SD conditions from the age of 14 weeks and left to acclimatize for 5 weeks (*n* = 12 per photoperiod) (Figure 1). At 19.5 weeks, all animals were injected with 30 mg/kg of alizarin, and this was repeated at 21 weeks. 3 days later, each animal received an osmotic mini-pump containing either vehicle or leptin (*n* = 12 per treatment split evenly per photoperiod). Animals were randomly assigned pumps although it was ensured that each cage housed at least one animal with vehicle treatment and one animal with leptin. At 24 weeks, the animals were injected with 20 mg/kg of calcein, which was repeated at 25.5 weeks. Both dyes were diluted in 0.01 M PBS and injected subcutaneously. Both dyes were administered at lower concentrations than study 2 to prevent potential distress to the animal by administering the larger volumes needed for the larger animal size. The animals were euthanized 3 days after the second calcein injection using sodium pentobarbital (Euthatal, Merial, UK). The ovaries and fallopian tubes and the retroperitoneal fat pads were removed from each animal and weighed. Femurs were removed for cortical appositional growth analysis, growth plate histology, and microCT analysis. Serum samples were obtained for analysis of leptin and bone markers.

### Administration of Leptin

Alzet osmotic mini-pumps (2004 model, Charles River, UK) were used to deliver recombinant mouse leptin (R&D Systems, UK) diluted in 20 mM Tris/HCl (pH 8). Animals were anesthetized under isoflurane (2–3%), and the mini-pumps were inserted subcutaneously in the flank region. Each mini-pump delivered 15 μg/day leptin over a 28-day period, a concentration previously shown to increase the serum leptin levels of Siberian hamsters after 14 days of continuous treatment (23–25).

### Blood Serum Analysis

Blood samples were acquired *via* cardiac puncture, and the plasma supernatant was collected and stored at −80°C. Serum leptin concentration was measured using a commercial “sandwich” ELISA (Mouse Leptin ELISA kit; Millipore, USA). The resulting absorbance was read at 450 and 590 nm using a multiskan spectrum spectrophotometer (Thermo Scientific, USA). The difference between the two readings was interpolated from a curve of the known leptin standards. The quality controls were also interpolated from the curve and fell within the acceptable range.

25-Hydroxy vitamin D_3_ analysis by mass spectrometry was kindly undertaken at the Department of Clinical Biochemistry at Norfolk and Norwich University Hospital using a Micromass Quattro Ultima with Masslynx acquisition software (Waters, USA) and their standard operating procedure.

Blood serum was analyzed for levels of interleukin-6 (IL-6), adrenocorticotropic hormone (ACTH), insulin, receptor activator of nuclear factor kappa-B ligand (RANKL), leptin, and tumor necrosis factor α (TNFα). This was completed using a Milliplex map mouse bone panel 2A (Millipore, USA) assay kit. The plate was read using a MAGPIX analyzer (Luminex, USA), and sample values were interpolated from known standards using the built-in xPONENT software.

### Appositional Growth

Bone samples, including femora and vertebrae, were fixed in 4% paraformaldehyde and embedded in JB4 resin (Agar Scientific, UK) for sectioning. Samples were dehydrated through increasing grades of ethanol (50, 70, and 90%), being in each concentration for 24 h. They were transferred to infiltrating resin and placed on a rocker for 48 h, with three changes of the resin during this time period. The samples were orientated in plastic molds and covered in embedding resin, which was polymerized in an anaerobic environment using anaerocult bags and sachets (Merck, UK) at 4°C overnight. The samples were sectioned at 10 µm onto gelatin-coated slides using a Leica supercut 2050 microtome.

Sections were imaged using a Leica DMRB fluorescence microscope with a Hamamatsu C4742-95 camera and Openlab (Perkin Elmer, USA) acquisition software. Images were acquired where bands of alizarin (red) and calcein (green) were observed next to each other in the cortical bone of the shaft of the long bones and the vertebral body of the lumbar vertebrae. Images were analyzed by measuring the distance between the bands using ImageJ software (National Institutes of Health, USA). A grid of crosses (1.17 cm^2^ per point) was laid over the image and where a grid point met one of the fluorescent bands, the distance measured was from that point to the nearest point on the next band. A minimum of 10 measurements were recorded for each bone, and the final value was an average of these measurements.

### Growth Plate

One femur from each animal was fixed in 4% paraformaldehyde for 48 h, decalcified in 10% EDTA in 0.1 M Tris (pH 7.4), and snap-frozen in isopentane (supercooled in liquid nitrogen). The samples were sectioned on a Leica CM1850 cryostat at 10 µm onto gelatin coated slides and were stained with Harris hematoxylin and 1% eosin (EMS, USA). The sections were imaged using a Leica DM4000B light microscope using a QImaging MicroPublisher RTV 3.3 camera and Openlab (Perkin Elmer, USA) acquisition software. The widths of proliferative and hypertrophic zones in clearly defined columns of the growth plate were measured using ImageJ software (National Institutes of Health, USA). 4–6 measurements were recorded per zone for each animal.

### MicroCT

**Figure 2:**
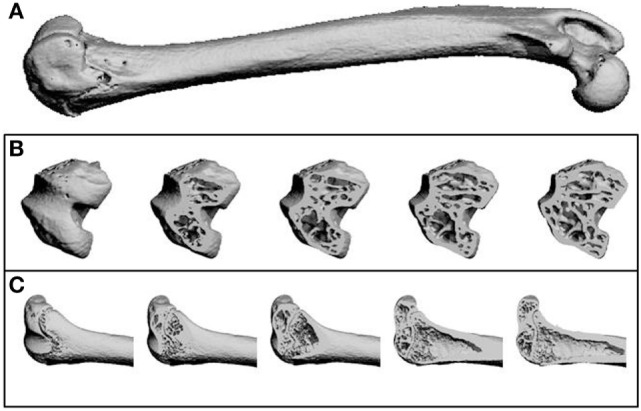
Representative microCT images as reconstructed by the Scanco software. **(A)** A whole femur. **(B)** Transverse sections through the femur from the distal (knee) end, showing trabecular structure. **(C)** Longitudinal sections through the femur at the distal (knee) end, showing trabecular structure and the growth plate.

MicroCT was carried out using one femur from each animal, which were embedded in resin as described above. Samples were scanned using a Scanco μCT40 (beam energy: 55 kVp, beam intensity: 145 µA, 200 ms integration time, and spatial resolution: 10 µm). Image reconstruction and analysis were performed using Scanco software (Figure 2).

### Statistics

Sample sizes were selected for studies 1 and 2 such that these experiments were sufficiently powered to detect a significant effect of photoperiodic treatment on body weight at 5 weeks and 6 months of age, respectively. For study 3, group sizes were the same as those previously used by Atcha et al. (25) who demonstrated that leptin infusions (15 μg/day) caused a significant reduction in adiposity in Siberian hamsters maintained in short days.

Data were analyzed by linear regression, unpaired *t*-test, one-way ANOVA and two-way ANOVA with Tukey’s *post hoc* test using Prism software (GraphPad, USA). Data are presented as mean ± SD.

## Results

### Study 1—The Effect of Photoperiod on the MAR of Juvenile (5-Week Olds) Siberian Hamsters

**Figure 3:**
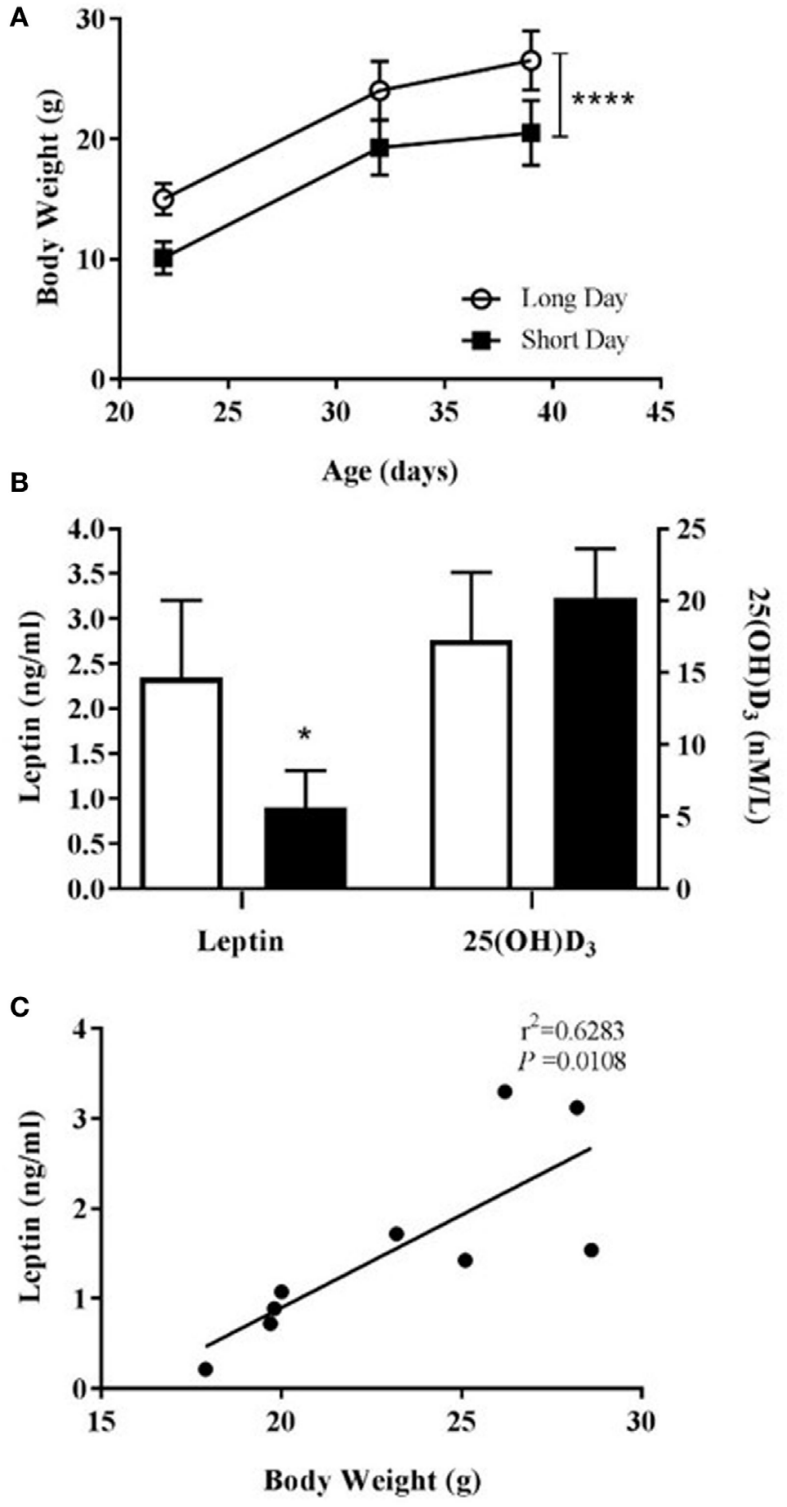
Physiological measurements of juvenile (5-week olds) Siberian hamsters born and weaned in long day (LD) and short day (SD) photoperiods. **(A)** Animal body weights recorded from 21 to 39 days old. **(B)** Serum levels of leptin and 25(OH)D_3_ from blood collected at 39 days old. Data are mean ± SD for *n* = 4 LD and *n* = 5 SD animals. ****P < 0.0001, two-way ANOVA; *P < 0.05, unpaired *t*-test. **(C)** Correlation between body weight and serum leptin level (*n* = 9, LD and SD animals combined), linear regression applied to data from individual animals.

Siberian hamsters were housed in either LD or SD photoperiods from confirmation of pregnancy. Those born and housed post-natally in SD were significantly lighter than those in LD at all points until the termination of the study at day 39 (*P* < 0.0001, Figure 3A). Animals housed in SD had significantly decreased blood serum leptin levels compared to LD (*P* = 0.0124, Figure 3B), and leptin levels were positively correlated with body weight within the whole cohort (*r*^2^ = 0.6283, *P* = 0.0108; Figure 3C). There was no difference in blood serum levels of vitamin D_3_ between the photoperiod groups at the end of the study (Figure 3B).

**Figure 4:**
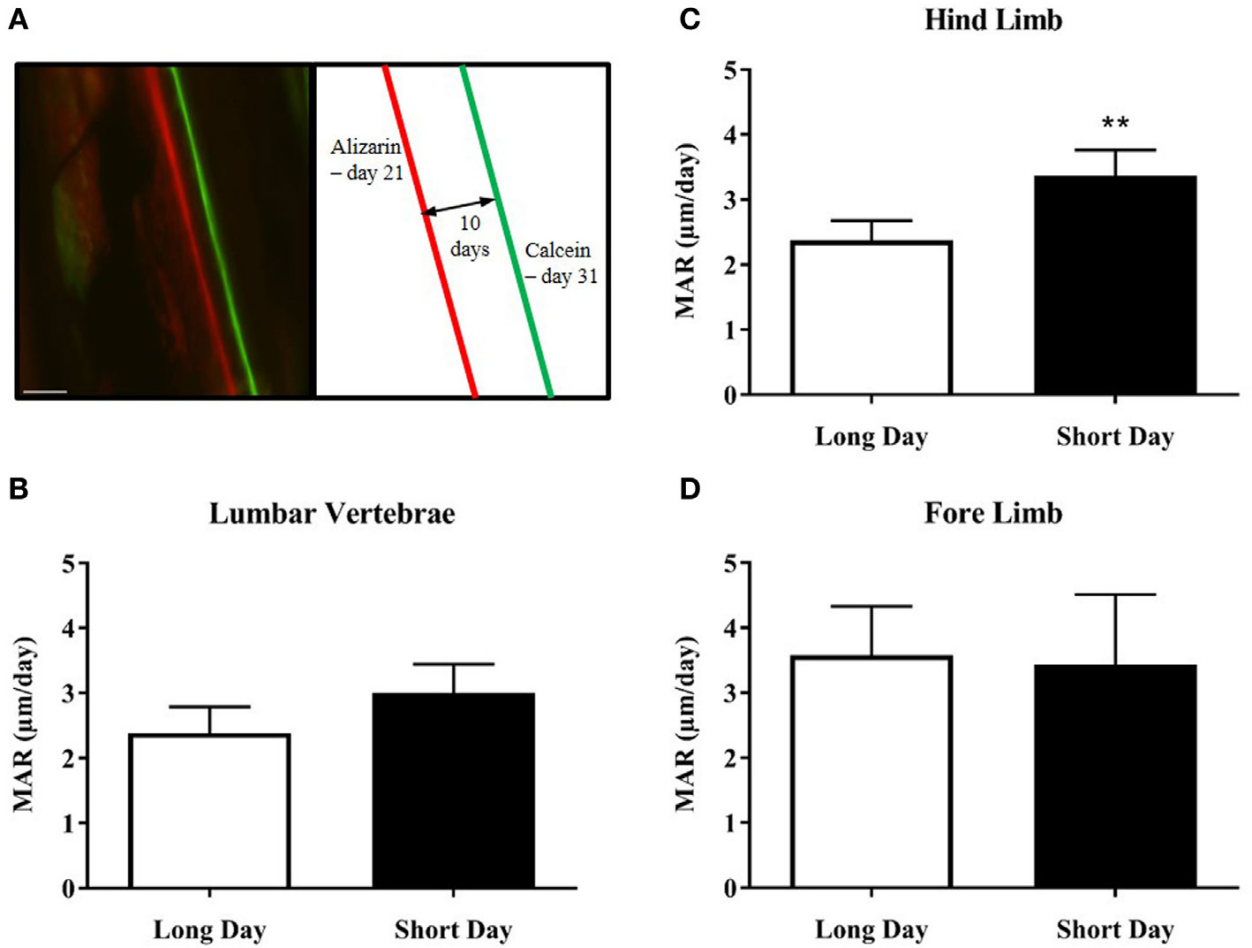
Effect of photoperiod on the mineral apposition rate (MAR) of lumbar vertebrae and long bones of juvenile (5-week olds) Siberian hamsters. **(A)** Representative double-labeled image showing the alizarin and calcein incorporation into the cortical bone of the femur, with an experimental schematic. Scale bar = 100 µm. **(B)** MAR of lumbar vertebrae, **(C)** hind limb long bones, and **(D)** fore limb long bones measured over 10 days (21–31 days old). Data are mean ± SD from *n* = 4 LD and *n* = 5 SD animals. **P < 0.01, unpaired *t*-test.

The hamsters were injected with calcium chelating dyes to label the mineralizing front in the cortical bone of their skeletons (Figure 4A). The distance between the labels was measured as growth over time and known as the MAR. Bone samples from both the axial and appendicular skeleton were analyzed. A photoperiodic difference was observed in the hind limb bones (femur and tibia), which showed an increased MAR in SD animals compared to LD animals (*P* = 0.0045, Figure 4C). There was no difference seen in the MAR of the lumbar vertebrae (Figure 4B) or the fore limb bones (Figure 4D).

### Study 2—The Effect of Photoperiod on the MAR of Adult (6-Month Olds) Siberian Hamsters

**Figure 5:**
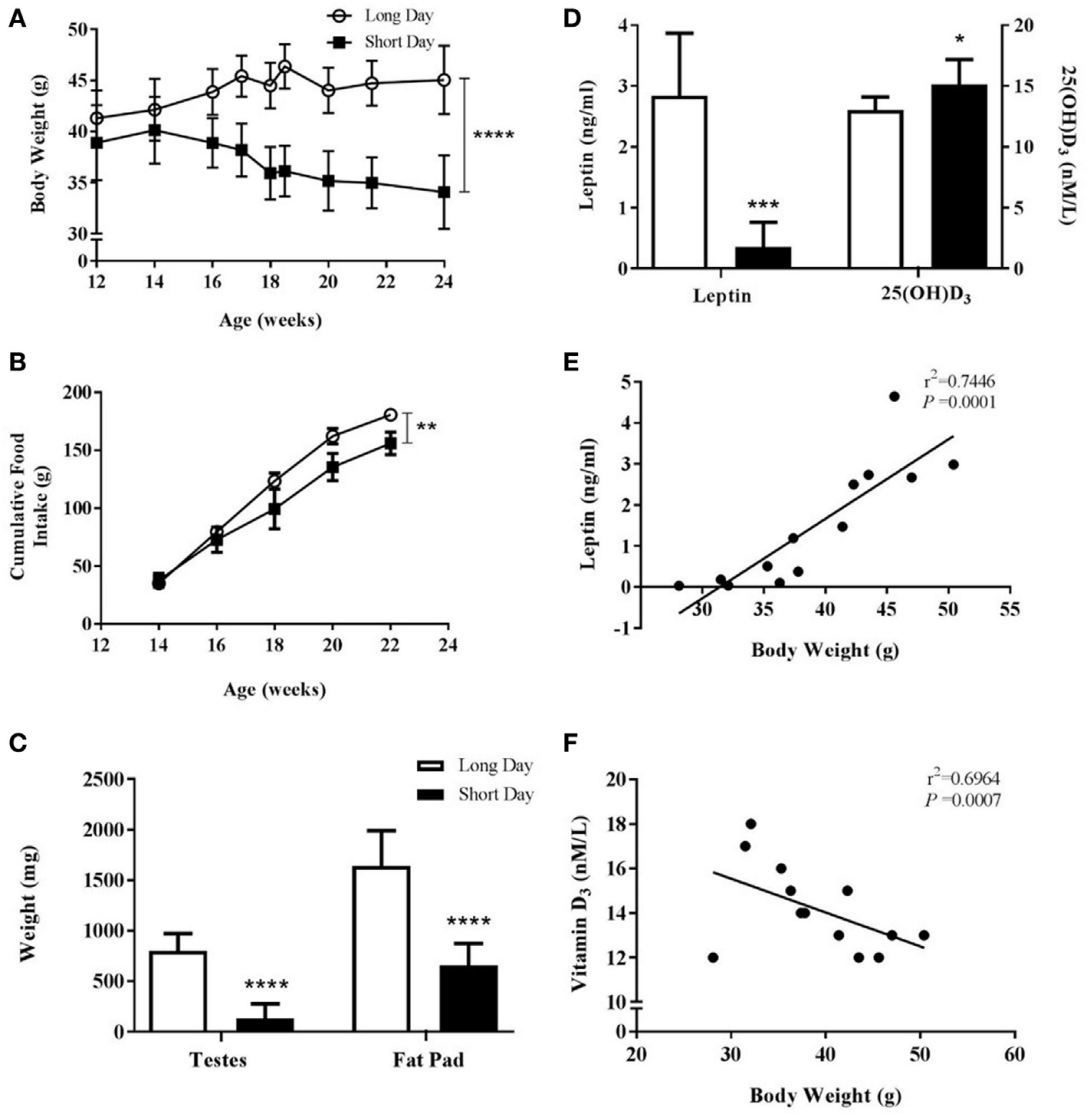
Physiological measurements of adult (6-month olds) male Siberian hamsters housed in long day (LD) and short day (SD) photoperiods. **(A)** Body weight recorded at least every 2 weeks from 12 to 24 weeks old. **(B)** Food intake displayed as a cumulative average per cage (*n* = 2 LD and *n* = 3 SD cages, 2–4 animals per cage). **(C)** Paired testes and epididymal fat pads were removed at 24 weeks old and weighed. **(D)** Serum leptin and 25(OH)D_3_ levels were measured in blood taken at 24 weeks old. Data are mean ± SD of *n* = 6 LD and *n* = 7 SD, unless otherwise stated. *P < 0.05, **P < 0.01, ***P < 0.001, ****P < 0.0001, **(A,B)** two-way ANOVA; **(C,D)** unpaired *t*-test. **(E)** Correlation between 24-week-old body weight and serum leptin level and **(F)** correlation between 24-week-old body weight and serum vitamin D_3_. Linear regression applied to data from individual animals.

To investigate MAR in older Siberian hamsters over a longer time period, male hamsters gestated and raised in LD were placed in either LD or SD environments at 12 weeks of age and monitored for a further 12 weeks. SD-housed Siberian hamsters had significantly decreased body weights compared to those in LD (*P* < 0.0001, Figure 5A) and had significantly reduced food intake over the course of the study (*P* = 0.0014, Figure 5B). The animals housed in SD showed reproductive regression and reduced adiposity, as measured by the wet weights of testes and fat pads, respectively (*P* < 0.0001, Figure 5C). Blood serum leptin levels were reduced in the SD animals (*P* = 0.0001, Figure 5D), and leptin levels were positively correlated with body weight within the whole cohort (*r*^2^ = 0.7446, *P* = 0.0001, Figure 5E). Blood serum 25(OH) D_3_ levels were increased in SD animals (*P* < 0.05, Figure 5D), and these levels were negatively correlated with body weight within the whole cohort (*r*^2^ = 0.6964, *P* = 0.0007, Figure 5F).

**Figure 6:**
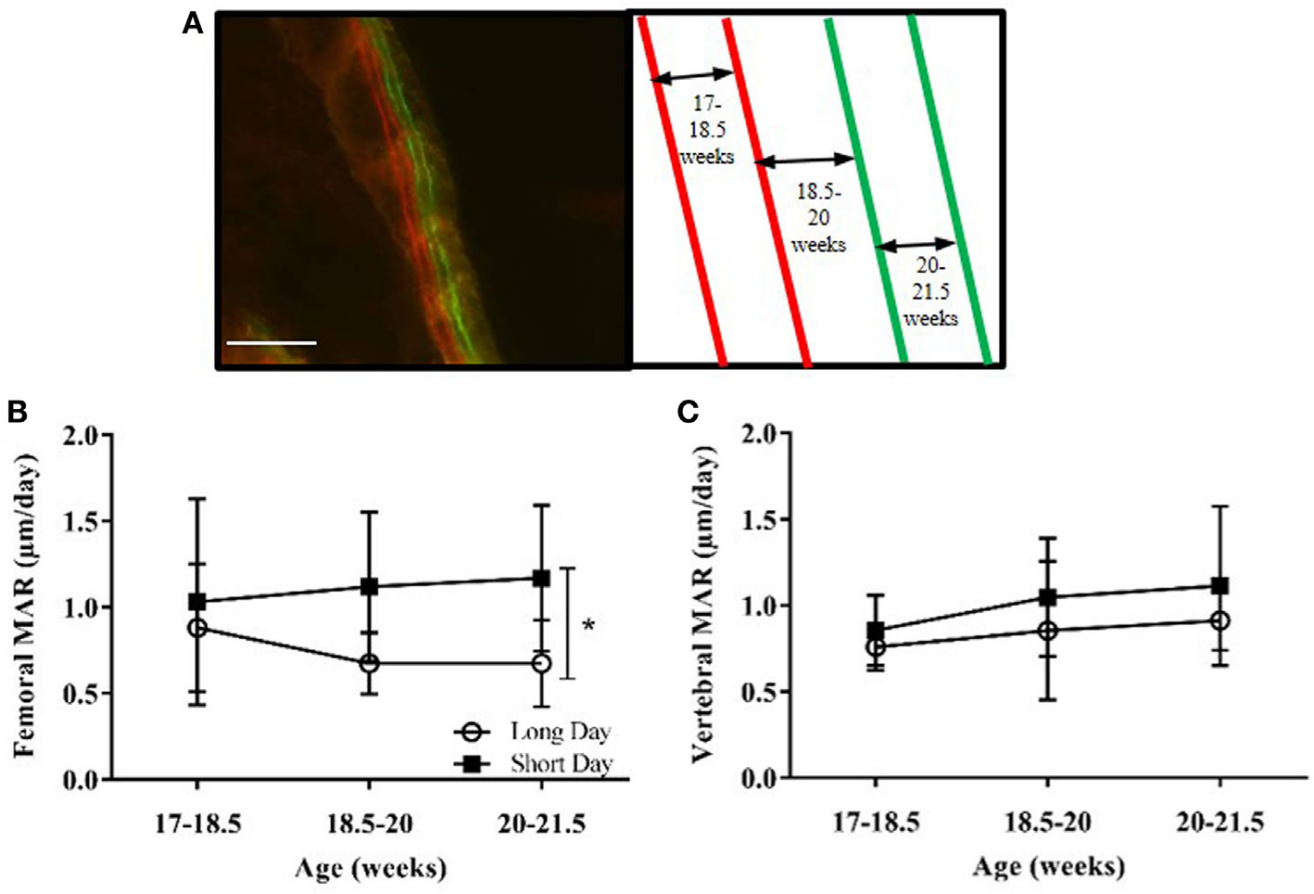
Effect of photoperiod on the mineral apposition rate (MAR) of femurs and lumbar vertebrae of adult (6-month olds) male Siberian hamsters. **(A)** Representative labeled image showing the double alizarin and double calcein incorporation into the cortical bone of the femur, with an experimental schematic. Scale bar = 100 µm. **(B)** MAR of femurs and **(C)** lumbar vertebrae measured at three intervals over 4.5 weeks. Data are mean ± SD, *n* = 6. *P < 0.05, two-way ANOVA.

Based on results from the first study, MAR was measured in the cortical bone of the femurs and the lumbar vertebrae. Calcium chelating dyes were injected at four time points to give three measurements of growth over time (Figure 6A). SD-housed hamsters had a significant increase in femoral MAR compared to LD animals (*P* = 0.0106, Figure 6B), but there was no difference in vertebral MAR between the two groups at any time point (Figure 6C).

### Study 3—The Effect of Leptin Treatment on the MAR, Growth Plate Size, and Bone Architecture of Adult Siberian Hamsters

**Figure 7:**
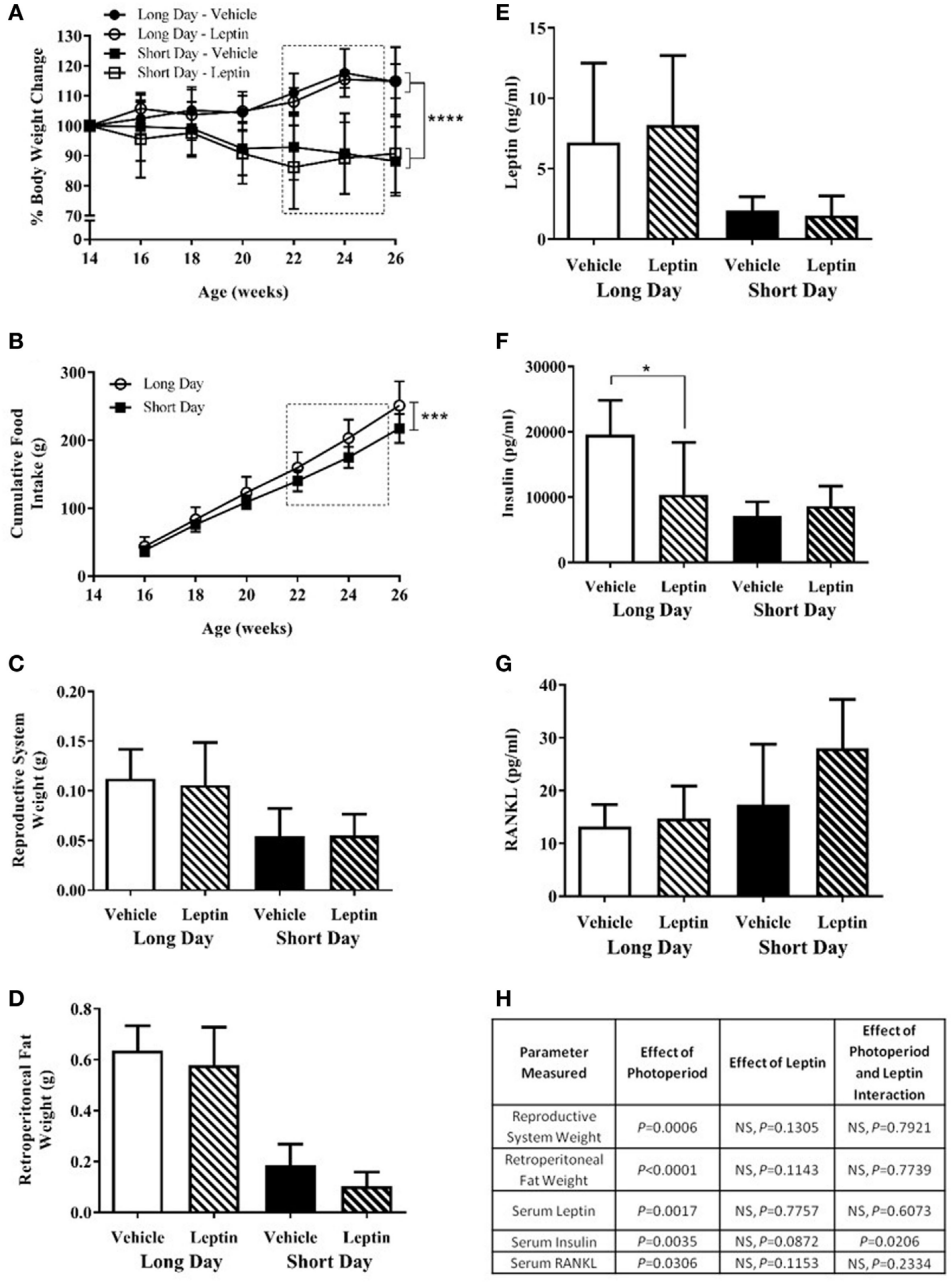
Physiological measurements of adult (6-month olds) female Siberian hamsters housed in long day (LD) and short day (SD) photoperiods and treated with vehicle (20 mM Tris/HCl) or leptin (15 μg/day). **(A)** Body weight recorded every 2 weeks from 14 to 26 weeks old. **(B)** Food intake displayed as a cumulative average per cage (*n* = 6 cages, 1–3 animals per cage). **(C)** Reproductive system (ovaries and fallopian tubes) and **(D)** retroperitoneal fat pads were removed at 26 weeks old and weighed. **(E)** Serum leptin, **(F)** insulin, and **(G)** RANKL levels were measured in blood taken at 26 weeks old. Data are mean ± SD, *n* = 6 (*n* = 5, long day—vehicle). *P < 0.05, ***P < 0.001, and ****P < 0.0001, **(A,B)** two-way ANOVA; **(C–G)** two-way ANOVA with Tukey’s *post hoc* test, with statistical data shown in **(H)**, NS—not significant. Box represents the period of vehicle/leptin treatment.

Study 3 was designed to investigate whether the low blood serum leptin levels observed in SD-housed hamsters contributed to the increased femoral cortical MAR observed in study 2. Animals raised in LD were transferred to SD photoperiods at 14 weeks of age or remained in LD, and at 22 weeks of age were treated with vehicle or leptin for 28 days *via* an osmotic mini-pump. Hamsters were treated with 15 μg/day leptin, a dose that had previously been shown to increase the blood serum leptin concentration of SD animals to levels comparable with LD animals (23, 24). Animals housed in the SD photoperiod had decreased body weights compared to those in LD (*P* < 0.0001, Figure 7A). There was no significant effect of leptin treatment on body weight in hamsters in either photoperiod (Figure 7A). SD-housed hamsters had decreased food intake over time compared to LD animals (*P* = 0.0003, Figure 7B). To avoid cage bias, animals from both treatment groups were housed together in the same photoperiod cage. Therefore, food intake data could only be compared for the photoperiod groups and not individual treatment groups.

Animals housed in the SD photoperiod had significant regression of their reproductive systems as assessed by the wet weight of ovaries and uteri and less retroperitoneal fat than those housed in LD (*P* = 0.0006, Figures 7C,H and *P* < 0.0001, Figures 7D,H respectively). There was no significant effect of leptin treatment on these parameters in either photoperiod.

Siberian hamsters housed in SD conditions had decreased blood serum levels of leptin (*P* = 0.0017, Figures 7E,H) and insulin (*P* = 0.0035, Figures 7F,H). There was a significant interaction of photoperiod and leptin on insulin levels (*P* = 0.0206, Figure 7H); *post hoc* tests revealed that insulin levels were decreased in the leptin-treated animals in LD but not in SD (*P* < 0.05, Figure 7F). An overall effect of photoperiod on serum RANKL levels indicated that levels were higher in hamsters in SD; leptin treatment did not affect this (Figures 7G,H). There was no difference in the ACTH levels of any photoperiod or treatment group, and the bone panel assay did not detect the presence of IL-6 and TNFα in the blood serum samples (data not shown).

**Figure 8:**
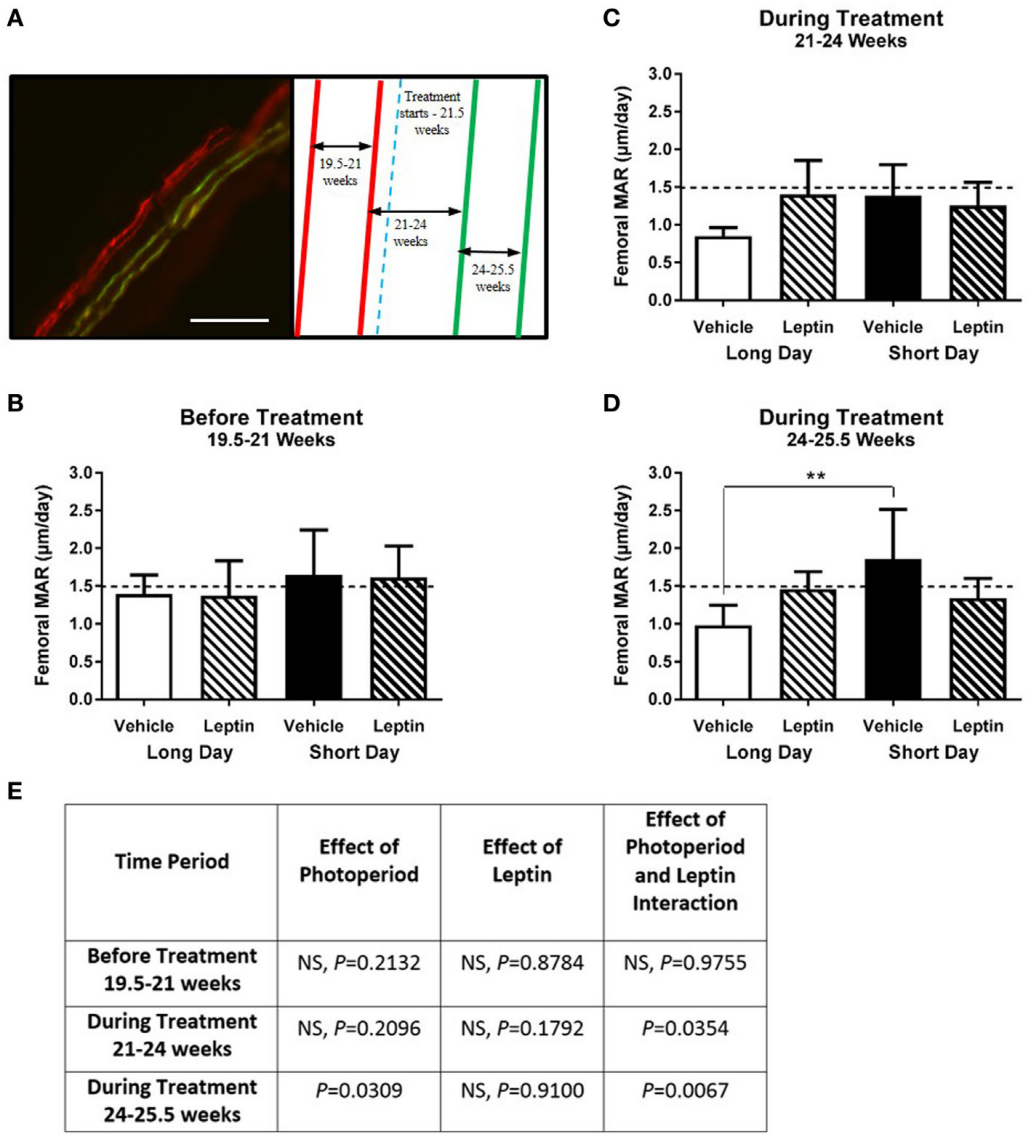
Effect of photoperiod and vehicle (20 mM Tris/HCl) or leptin (15μg/day) treatment on the femoral mineral apposition rate (MAR) of adult (6-month olds) female Siberian hamsters. **(A)** Representative labeled image showing the double alizarin and double calcein incorporation into the cortical bone of the femur, with an experimental schematic showing the point at which vehicle or leptin treatment began. Scale bar = 100 µm. MAR of femurs measured at **(B)** 19.5–21 weeks—before treatment, **(C)** 21–24 weeks—during treatment, and **(D)** 24–25.5 weeks—during treatment. Data are mean ± SD, *n* = 6 (*n* = 5, LD—vehicle). **P < 0.01, two-way ANOVA with Tukey’s *post hoc* test, with statistical data shown in **(E)**, NS—not significant.

Calcium chelating dyes were injected to assess femoral cortical MAR both before and during the treatment period (Figure 8A). Before treatment, there was no significant effect of photoperiod on MAR in femurs (Figures 8B,E). During the first period of treatment (21–24 weeks of age) there was a significant interaction of leptin and photoperiod (*P* = 0.0354, Figures 8C,E), but no main effect of the variables individually. During the second period of treatment (24–25.5 weeks), the animals housed in the LD photoperiod had a decreased femoral MAR compared to SD hamsters (*P* = 0.0309). There was a very significant interaction of leptin and photoperiod (*P* = 0.0067, Figures 8D,E), thus MAR was increased in leptin-treated hamsters in LD, but reduced in those in SD (Figure 8E).

**Figure 9:**
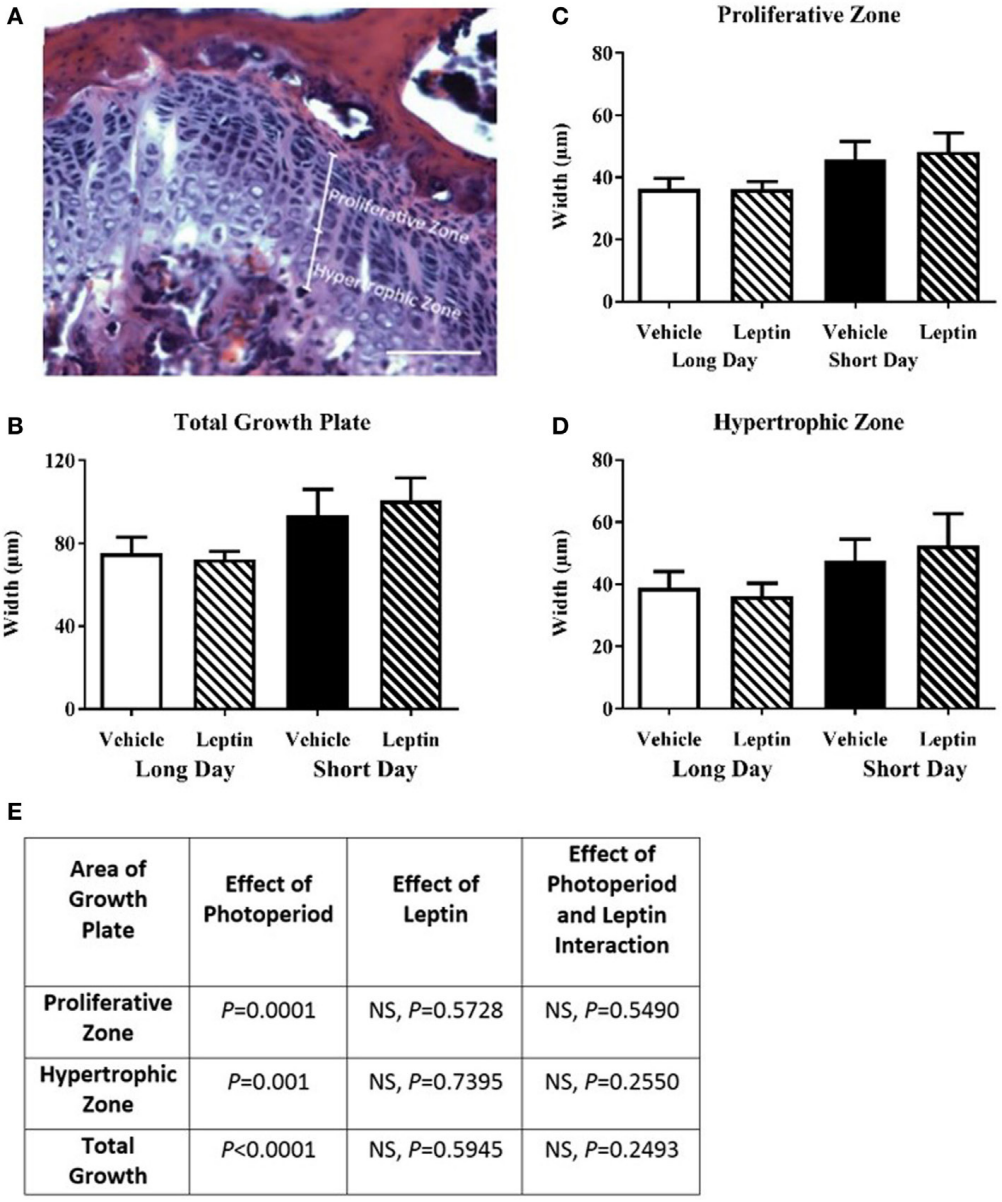
Effect of photoperiod and vehicle (20 mM Tris/HCl) or leptin (15 μg/day) treatment on the femoral growth plate width of adult (6-month olds) female Siberian hamsters. **(A)** Representative decalcified, H&E-stained growth plate showing measurement of proliferative and hypertrophic zones, scale bar = 100 µm. **(B)** Total growth plate, **(C)** proliferative zone, and **(D)** hypertrophic zone widths, measured where clearly defined columns were visible. Data are mean ± SD, *n* = 5. Two-way ANOVA with Tukey’s *post hoc* test, with statistical data shown in **(E)**, NS—not significant.

Femoral growth plates were labeled with hematoxylin and eosin in decalcified frozen sections. The proliferative and hypertrophic zone widths of a column were measured and recorded individually along with the total width of the two zones combined (Figure 9A). Overall, the growth plates of the SD housed hamsters were wider than those housed in LD, with a significant increase being observed in the total growth plate (*P* < 0.0001, Figures 9B,E). This significant effect of photoperiod was evident when the proliferative (*P* = 0.0001, Figures 9C,E) and hypertrophic zones (*P* = 0.001, Figures 9D,E) were analyzed separately. There was no effect of leptin treatment on the width of any of the zones measured.

MicroCT analysis of the femurs was carried out to detect cortical and trabecular bone differences within the treatment groups. There was a significant increase in bone volume of the SD hamsters’ tibias compared to the LD housed animals (*P* = 0.02), but no significant effect of leptin treatment. There were no differences in the other parameters measured, including bone surface, density and number, spacing and thickness of the trabeculae (Table 1).

**Table 1:** Effect of leptin treatment on femoral bone density of female adult Siberian hamsters housed in long day and short day photoperiods.

Parameter measured	Long day vehicle	Long day leptin	Short day vehicle	Short day leptin	Effect of photoperiod	Effect of leptin	Effect of photoperiod and leptin interaction
Bone volume (mm^3^)	21.27 ± 0.84	19.37 ± 2.00	22.35 ± 0.70	22.07 ± 0.96	*P* = 0.02	NS, *P* = 0.1370	NS, *P* = 0.2577
Bone surface (mm^2^)	218.1 ± 20.82	216.3 ± 7.72	228.3 ± 11.23	226.6 ± 9.50	NS, *P* = 0.2333	NS, *P* = 0.8311	NS, *P* = 0.9997
Bone surface/bone volume ratio	10.12 ± 1.03	11.06 ± 1.01	10.08 ± 0.78	10.12 ± 0.66	NS, *P* = 0.3562	NS, *P* = 0.3552	NS, *P* = 0.3952
Mean bone density (1/cm)	2.593 ± 0.05	2.560 ± 0.05	2.511 ± 0.09	2.559 ± 0.10	NS, *P* = 0.3403	NS, *P* = 0.8684	NS, *P* = 0.3526
Trabecular number (1/mm)	1.479 ± 0.74	0.9993 ± 0.47	0.9812 ± 0.75	1.686 ± 0.28	NS, *P* = 0.7867	NS, *P* = 0.7473	NS, *P* = 0.1157
Trabecular spacing (mm)	1.018 ± 0.94	1.403 ± 0.62	0.8327 ± 0.77	0.6067 ± 0.13	NS, *P* = 0.2521	NS, *P* = 0.8465	NS, *P* = 0.4650
Trabecular thickness (mm)	0.2420 ± 0.02	0.2171 ± 0.02	0.3218 ± 0.14	0.2419 ± 0.02	NS, *P* = 0.2113	NS, *P* = 0.2107	NS, *P* = 0.4970
Connectivity density (mm^3^)	1.503 ± 0.62	1.382 ± 0.33	1.361 ± 0.51	1.223 ± 0.16	NS, *P* = 0.5775	NS, *P* = 0.6292	NS, *P* = 0.9753

*Data given as mean ± SD, *n* = 3 (*n* = 4, long day—vehicle). Two-way ANOVA with Tukey’s *post hoc* test, NS—not significant*.

## Discussion

Our data demonstrate a significant effect of photoperiod on indicators of bone growth in Siberian hamsters. This study investigated appositional bone growth, *via* MAR, and measured longitudinal bone growth at the cartilage growth plate and found both to be increased in SD housed animals. We further investigated leptin as a potential mediator of this effect and found it to have an anti-osteogenic effect on MAR but no effect on growth plate width.

The Siberian hamster model is well established as a means of examining the effects of photoperiod on energy balance. We found that SD animals had decreased food intake, body weight, and associated decreases in body fat, despite being fed *ad libitum*, consistent with many reports in the literature [see Ref. (16, 17) for review]. In addition, both male and female SD animals underwent reproductive system regression, and we observed seasonal changes in pelage. Importantly, we found SD animals to have significantly lower serum leptin levels than their LD counterparts. Further serum analysis showed a significantly increased serum vitamin D level in the SD state, which considering their reduced food intake and light exposure might not have been predicted. This may be due, however, to the fat-soluble nature of vitamin D which may have been sequestered in adipose tissue and released as abdominal fat reserves are catabolized in SD (26, 27).

The seasonal change in leptin observed in rodent models has rarely been investigated in relation to its effect on bone; the single study in this area found no effect of a 1 week leptin supplementation on bone mass or mechanical properties, but did not investigate bone growth (23). The present study investigated appositional bone growth *via* MAR and measured longitudinal bone growth at the cartilage growth plate in addition to bone density studies. We have shown a significant and consistent increase in MAR of SD animals independent of gender and age in the cortical bone of the hind limbs (tibias and femora). This study allowed us for the first time to also analyze chondrocyte responses to photoperiod in the growth plates. We investigated growth plate size in 6-month-old female animals and found a significant increase in total growth plate width in the SD state and specific increases in the proliferative and hypertrophic zones of animals. An initial study of bone mass at 5 weeks of age and a more comprehensive study of tibial bone mass parameters at 6 months of age showed no significant difference (data not shown). An increase in total bone volume in SD hamsters was observed; however, this is more attributed to an increase in bone growth than bone density. These bone density data are consistent with the previous study (23) that used the same animal model at similar ages and leptin dose levels, but with a shorter duration of leptin supplementation: the present study treated hamsters for 4 weeks beginning at 22 weeks of age compared to 2 weeks beginning at 24 weeks of age. We have added significantly to the findings of Rousseau et al (23) by showing a reproducible negative effect of the LD higher leptin state on parameters of bone growth.

Although our results suggest decreased levels of serum leptin are beneficial to bone growth, other factors that could potentially affect bone growth also differ between the photoperiod states. In the SD state, Siberian hamsters undergo regression of the reproductive system, and as would be expected this is associated with a decrease in circulating levels of estradiol in female hamsters (21). Additionally, in male hamsters, testosterone levels are decreased in SD-housed animals (22), meaning there is less available for aromatization to estradiol. The decrease in circulating estrogens in women after menopause is strongly linked to the higher bone turnover rate and subsequent bone loss observed in post-menopausal osteoporosis, with estradiol treatment reversing the phenotype in ovariectomized animals. As such, it would be expected that the lower sex hormone levels would have a negative effect on the bone metabolism of SD-housed animals and that this would be observed in their bone density measurements. As this was not the case for either sex, it is unlikely that this factor contributed to the changes in bone growth rate seen in the SD state.

The nocturnal duration of pineal melatonin secretion increases in SD-housed Siberian hamsters (28), and this prompts the development of the SD phenotype through actions in the pars tuberalis and hypothalamus (18). Melatonin has been suggested to have a positive direct effect on bone growth, although the mechanism has not been clearly established. Satomura et al. (29) observed an increase in murine femoral MAR after treatment with melatonin, but in contrast, Koyama et al. (30) found that although melatonin had positive effects on bone density in mice, it had no effect on MAR. The differences seen were hypothesized to be due to a decrease in resorption rather than an increase in bone formation, despite other groups finding melatonin increases human osteoblast proliferation and production of differentiation markers *in vitro* (29, 31). The currently published literature demonstrates that melatonin has an impact on MAR, and therefore, could be a factor contributing to the increased cortical MAR observed in this study. Unfortunately, no direct comparison can be made, due to the melatonin treatment in these studies being at a supraphysiological level.

25-Hydroxy vitamin D_3_ levels differed between the photoperiod groups and were increased in SD animals where body fat was reduced, but the impact of increased vitamin D is reported as both positive and negative. For example, human studies have shown vitamin D_3_ supplementation to have a positive effect on bone mineral density in adult girls (32), and serum levels to be positively correlated with bone mineral density of young and old people (33). In contrast, *in vitro* studies with human and animal bone cells have shown vitamin D_3_ to have negative effects on cell proliferation and differentiation (34, 35). In addition, murine osteoblasts lacking the vitamin D receptor had increased proliferation and increased early and late differentiation markers mineralization (36). These contrasting findings may be due to the role of vitamin D in both increasing bone resorption to release calcium reserves from bone and encouraging bone formation at times of excess calcium availability. As vitamin D levels were increased in the present study in the SD phenotype, vitamin D could have contributed to the increased MAR seen; however, its effect remains unknown.

Given that factors other than leptin have potentially influenced the increased MAR seen in SD animals, a second set of studies was carried out to understand better the potential role of leptin. Siberian hamsters exposed to SD were treated peripherally with murine leptin at a dose level designed to restore LD concentrations of leptin. Previous studies showed that peripheral treatment of 15 μg/day leptin over 2 weeks increased the serum leptin levels of a SD animal to that of a hamster housed in the LD environment (23–25). A strength of the present study was the ability to monitor MAR changes longitudinally, and it was therefore designed to measure MAR before treatment started and again after 2 weeks of treatment, at which point the leptin levels of the SD treated and LD vehicle animals would be expected to be similar.

The same robust effect of SD photoperiod on body weight, food intake, reproductive regression, fat weight, and serum leptin levels was observed. However, there was no difference in any of these parameters when comparing the vehicle and leptin-treated animals in either photoperiod. Food intake was only compared between photoperiods, as hamsters from different treatment groups were housed together. This is a potential limitation of the study; however, it was deemed that removing the potential bias of housing the treatment groups separately outweighed the gain of information from the food intake. In addition, it has been previously demonstrated that the change in body weight observed in leptin-treated Siberian hamsters can occur without a significant change in food intake (25). The failure to observe a metabolic response in LD animals has been observed in previous studies using this dose of leptin and is potentially due to a development of central resistance (23–25). Levels of bone markers had not previously been reported in these animals, and this study found that RANKL was increased in SD animals. This would imply an increase in osteoclastogenesis and therefore bone resorption; however, there were no observed differences in bone density and architecture. This is difficult to interpret in isolation, and investigation into the RANKL/OPG ratio would shed further light on the relevance of this finding. Insulin levels were decreased in the SD animals in agreement with Bartness et al. (37), and additionally, an interaction effect of leptin and photoperiod was observed. Leptin has been shown to decrease glucose-stimulated insulin secretion in a dose-dependent manner through central, rather than peripheral mechanisms (38). This adds further evidence to the suggestion of central leptin resistance occurring in the LD-housed animals, as their insulin levels decreased after treatment with leptin, implying that peripheral mechanisms were no longer effective.

Mineral apposition rate of the Siberian hamsters was significantly affected by leptin treatment, and interestingly, this effect differed depending on the photoperiod the animals were housed in. In agreement with the first two experiments, the MAR of SD animals was increased compared to LD in the vehicle animals, and so it was expected that leptin would have a negative effect on MAR in animals treated with recombinant leptin. However, rather than a consistent effect of leptin in animals housed in each photoperiod, there was an interaction effect between leptin treatment and photoperiod. This resulted in MAR being lower in SD animals treated with leptin and higher in LD animals, compared to their vehicle counterparts. This study was designed to manipulate the serum leptin levels of SD animals to be similar to LD hamsters. The MAR of SD-treated animals was decreased to the point that it was no longer significantly different to LD animals, supporting an anti-osteogenic role of leptin. A role for leptin in bone growth and remodeling has been reported in many studies; however, the results have been conflicting. Leptin knockout mouse models have been the basis of a majority of the *in vivo* studies and these showed leptin to have both osteogenic (6, 39–41) and anti-osteogenic properties (5, 42–44). In our initial studies, serum leptin levels were significantly lower in the SD animals, and so there is was potentially an association between decreased serum leptin levels and increased MAR. In agreement with this, increased MAR has been demonstrated in leptin-deficient, *ob/ob*, mice (43, 44). However, there is also contrasting data from Bartell et al. (45) who showed *ob/ob* mice to have increased MAR only after leptin treatment. Taken together, the results of the present study are all consistent with an anti-osteogenic role of leptin.

Leptin had an opposite effect on LD-treated animals, with MAR being higher than the vehicle animals. Although this was not expected, it lends further evidence to the suggestions of leptin resistance occurring in LD, leptin-treated animals (23–25). Human obesity has also been described as a state of leptin insensitivity, as high levels of food intake and body adiposity occur despite very elevated circulating leptin concentrations. A physiological resistance at the blood–brain barrier has been hypothesized as explanation for this (46). It has been demonstrated that leptin uptake into the brain is reduced in obese mice and control mice that are treated with excess leptin (47). This was despite no change in the expression of *Ob-Ra*, the receptor responsible for blood–brain barrier transport of leptin (48), indicating a mechanistic change that is yet to be defined. In agreement with this, the body weight reducing action of leptin has been demonstrated in obese rodents if treated centrally, but not peripherally (49). We also found serum insulin levels to be decreased in leptin-treated animals in LD, which may well reflect leptin modulating glucose homeostasis *via* central mechanisms (38), consistent with the idea that leptin could also act *via* a centrally mediated mechanism to negatively regulate appositional bone growth in our animal model.

We have reported femoral growth plate size measurements in Siberian hamsters for the first time and have shown that there is an effect of photoperiod. SD-housed animals had increased total growth plate width, and this was seen in both the proliferative and hypertrophic zones, consistent with the increased bone growth rates seen using MAR. In addition, this study found that the growth plate widths were not affected by leptin treatment. This is in contrast to several studies which found growth plate length to be increased after leptin treatment (39, 50, 51), and the long bones of *ob/ob* mice to be shorter (6, 39–41). Bone density measurements agreed with our initial studies, showing no measurable effect of photoperiod, or leptin, consistent with previously published observations (23).

A limitation of the current study was that the leptin delivered *via* the pump ceased prior to the study ending. In previous studies, animals were treated for 14 days before euthanasia using sequential implantation of two osmotic mini-pumps that were changed after 7 days (23, 24). In the present study, the hamsters received a single 28-day osmotic mini-pump to minimize surgical stress to the animals, but blood samples and tissues were not collected until day 31 after the implantation of mini-pumps. This is likely to have resulted in the observation that leptin levels were not significantly different between leptin and vehicle treatment groups within each photoperiod, as leptin has a half-life of less than an hour in rodents (52), so levels would rapidly revert to normal after supplementation ends (53). Unfortunately, it was not possible to measure leptin longitudinally due to the small blood volume of a Siberian hamster and difficulties in obtaining samples (no accessible tail vein). While it is recognized that the body weight changes and serum leptin levels at the end of the study are not as expected, there is evidence to show that bone changes due to leptin are apparent at much lower levels than those needed for changes in body weight (54). In addition, the *l/l* mouse model, which has increased leptin signaling, is osteoporotic despite no changes in food intake or reproductive function, suggesting that a smaller change in leptin signaling is required to affect bone compared to its other functions (55).

In conclusion, we have demonstrated for the first time that photoperiod has a reproducible and significant effect on bone growth parameters including both MAR and growth plate size at several time points and in both sexes. No changes were observed in measures of bone mass, consistent with the only previous study examining these parameters (23). While inconclusive, we observed some findings of interest that warrant further study. First, with leptin treatment the MAR of SD animals reduced and began to approach those of LD animals to the point where they was no longer a statistically significant difference between them, implying that leptin has a catabolic effect on bone growth. Second, the effects of leptin treatment were photoperiod-dependent, lacking the usual significant reduction in MAR observed in leptin-treated hamsters in LD. This supports the hypothesis that supplementing leptin in an existing state of adiposity can promote leptin resistance (probably centrally mediated, supported by the insulin findings), and further implies an anti-osteogenic role for leptin in bone.

## Ethics Statement

This study was carried out in accordance with the recommendations of the UK Animals (Scientific Procedures) Act of 1986 (project licenses: PPL 40/3065 and PPL 40/3604). The protocol was approved by the University of Nottingham Animal Welfare and Ethical Review Board.

## Author Contributions

Conception and design: MK, FE, and SA. Data collection: MK, FE, JH, and SA. Data analysis and interpretation: MK, FE, and SA. Drafting the article: MK and SA. Critical review of the article: MK, FE, JH, and SA. Final approval of the article: MK, FE, and SA.

## Conflict of Interest Statement

There are no financial or commercial relationships that present a potential conflict of interest. This research did not receive any specific grant from funding agencies in the public, commercial, or not-for-profit sectors.
